# Overhead Transmission Line Sag Estimation Using a Simple Optomechanical System with Chirped Fiber Bragg Gratings. Part 1: Preliminary Measurements

**DOI:** 10.3390/s18010309

**Published:** 2018-01-20

**Authors:** Michal Wydra, Piotr Kisala, Damian Harasim, Piotr Kacejko

**Affiliations:** 1 Department of Power Systems, Lublin University of Technology, 20-618 Lublin, Poland; p.kacejko@pollub.pl; 2 Institute of Electronics and Information Technology, Lublin University of Technology, 20-618 Lublin, Poland; d.harasim@pollub.pl (P.K.); p.kisala@pollub.pl (D.H.)

**Keywords:** power system monitoring, power lines sag measurement, chirped fiber Bragg gratings, quasi-periodic waveguide structures, strain measurement, elongation measurement

## Abstract

A method of measuring the power line wire sag using optical sensors that are insensitive to high electromagnetic fields was proposed. The advantage of this technique is that it is a non-invasive measurement of power line wire elongation using a unique optomechanical system. The proposed method replaces the sag of the power line wire with an extension of the control sample and then an expansion of the attached chirped fiber Bragg grating. This paper presents the results of the first measurements made on real aluminum-conducting steel-reinforced wire, frequently used for power line construction. It has been shown that the proper selection of the CFBG (chirped fiber Bragg grating) transducer and the appropriate choice of optical parameters of such a sensor will allow for high sensitivity of the line wire elongation and sag while reducing the sensitivity to the temperature. It has been shown that with a simple optomechanical system, a non-invasive measurement of the power line wire sag that is insensitive to temperature changes and the influence of high electromagnetic fields can be achieved.

## 1. Introduction

Fiber Bragg grating (FBG) sensors can be used to measure many physical quantities, such as strain, stress, elongation, temperature [1], and refractive index [2]. The advantage of optical method based research is the lack of influence on the tested object, the ability to perform non-invasive measurements which can be performed in a wide variety of fields such as mechanical structures diagnostics or even medicine [3]. Among the FBG structures are some significant gratings in which the period is not uniform [4]. Many studies have been published demonstrating the promising properties of periodic structures with non-uniform periods, especially chirped fiber Bragg gratings [5]. These structures can be used for separated parameter monitoring [6], independent strain and temperature measurement [5,7,8], dispersion compensation [9,10], or structural health monitoring [11]. Tapered fibers with CFBG have significantly improved strain sensitivity [12]. The CFBG sensors can also be used with inexpensive interrogators [13] and their specially-designed structures can be used as optical filters [14,15,16]. The loads of the overhead power transmission lines can be measured using a few uniform FBGs. In some embodiments, each grating is bonded directly to the conductor with epoxy [17], which makes it difficult to disassemble when the sensor head cracks. There are also known systems for distributed monitoring of overhead transmission lines with fiber Bragg grating sensor networks [18]. Such systems are very promising because they can be used to provide multiple types of information for smart grids. FBG sensors can be fairly used to monitor the strain of transmission line wire. In this case, the strain of the conductor is directly proportional to the ampacity of the cable [19]. In such systems, the strain of the grating cannot be widely adjusted because the optical fiber extends equally with the power line conductor. In this paper, the authors would like to present the construction of sensing heads where the CFBG is attached to the so-called control sample steel plate whose dimensional adjustment allows for the control of the sensitivity and measuring range. Among the various methods of constructing these periodic structure based sensors, this is the first method proposed to the best of our knowledge in which the CFBG can be implemented with the strain and sag of power lines can be measured using a special optomechanical system and the control specimen. Today, modern power systems and smart grids are required to have the operational flexibility of electrical power transmission whilst preserving the appropriate safety margin [20,21]. In the context of overhead transmission lines (OTL), the above approach can be implemented by dynamic line rating (DLR) systems [22,23,24,25]. The DLR systems allow for the continuous monitoring of operating conditions and the electromechanical state of the OTL span [20,26] or of the whole line [27]. The most important parameters determining the safe operation of the OTL, among others, are the sag and strain of the wire, which are direct functions of its actual temperature resulting from a load of electric current and atmospheric conditions [28,29,30]. The unquestionable advantages of fiber-optic and FBG sensors are the insensitivity of the measurement to the strong electromagnetic field, the speed of measurement, the non-invasive installation of the sensor on the conductor, the small power demand for the supply of the measuring system, and not needed a complex sensor or radio [31]. At present, the dynamic development of the FBG based monitoring systems is observed [32,33]. Using OTL monitoring and its actual state [28], the current wire elongation sag is a critical parameter in determining the distance from the ground or obstacle [34] that is affecting the operational safety.

This article aims to present the results of the application of the CFBG-based sensor for power line strain (relative elongation) to calculate its sag, as an alternative to the standard FBG sensors.

## 2. Materials and Methods 

**Figure 1:**
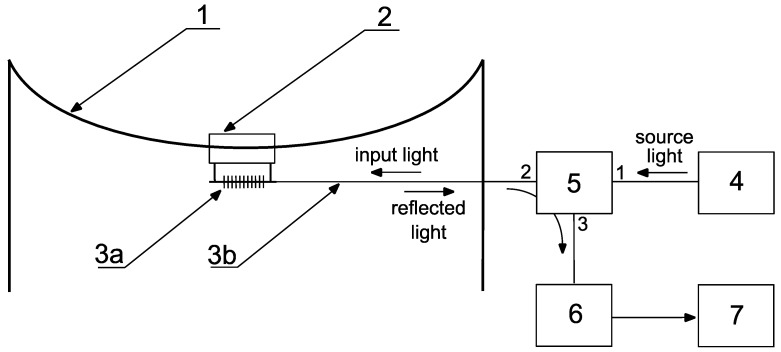
The overhead transmission line wire sag measurement system where: 1: the ACSR 26/7 Hawk conductor; 2: the measuring clamps/sensing head; 3a: the optical fiber; 3b: the inscribed chirped fiber Bragg grating; 4: the light source with a stabilized super-luminescent diode; 5: the optical circulator; 6: the optical spectrum analyzer; and 7: the computer/gateway.

The chirped gratings, compared to conventional FBGs where the period of the refractive index perturbations is constant along the grating length, are characterized by a variable period in the internal structure. Chirps in gratings may take many different forms. The period may vary linearly with the length of the grating, it may be quadratic, and so on. The chirp could also be symmetrical where the period varies by increasing or decreasing around the pitch in the middle of a grating. Generally, modifications applied to the internal structure of the gratings leads to a change of its spectral characteristic. The most widely used CFBGs have a widened spectrum which makes them possible to be used in many areas, including dispersion compensators, mirrors for specialized photonic sensing systems, and sensing elements replacing conventional FBGs. Their widened characteristics allow them to measure not only the wavelength shift, but also the width of the spectra [35]. The proposed method for the power line sag estimation utilizes a simple optomechanical system which allows for the line’s sag change to be converted to the CFBG optical parameter change. The idea of the system is presented in Figure 1. Similar solutions were presented in [17,18] where a sensing head was mounted on an energized power line conductor and the ground voltage was connected directly by fiber-optic cables in the light spectrum processing devices.

**Figure 2:**
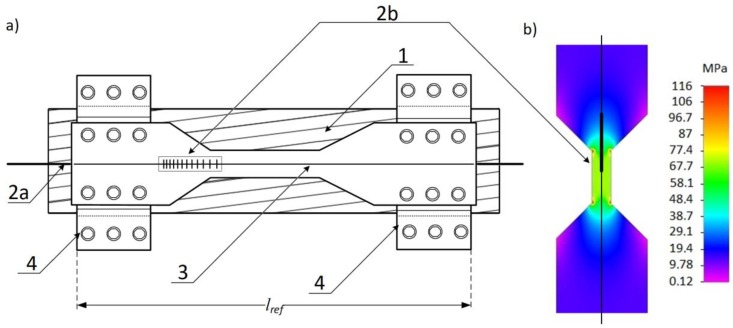
(**a**) the optomechanical system using a chirped fiber Bragg grating and a mechanical strain transformer where: 1: the ACSR 26/7 (Hawk) conductor; 2a: the optical fiber; 2b: the chirped fiber Bragg grating; 3: the thinned steel plate; 4: the semi-circular screwed clamps, *l_ref_*: reference sensing head length; and (**b**) the strain distribution in the proposed steel testing plate while extending the measurement section.

The system shown in Figure 1 allows for the elongation and sag measurement of the OTL wire. The light source was a superluminescent light emitting diode (SLED). The Thorlabs S5FC1005S (Thorlabs, Newton, NJ, USA) was attached to a single-mode optical fiber used as a transmission light-guide. The light emitted from the SLED source illuminated the sensing CFBG with a 1 nm/cm chirp, inscribed on the hydrogen-loaded SMF-28 part at the end of the fiber. The optical fiber with the CFBG sensor was connected/glued to a thinned plate which was 1 mm in thickness and made of steel (1.0037 S235JR UNI according to the norm EN 10025). The shape of the control plate is shown in Figure 2. The plate is screwed to the semi-circular clamps fixing the control plate with the CFBG sensor to an OTL wire (Figure 2). The elongation of the OTL wire causes the clamps to move apart and increase the tension of the control plate, which is monotonically extending the length of its measuring section with the CFBG attached.

**Figure 3:**
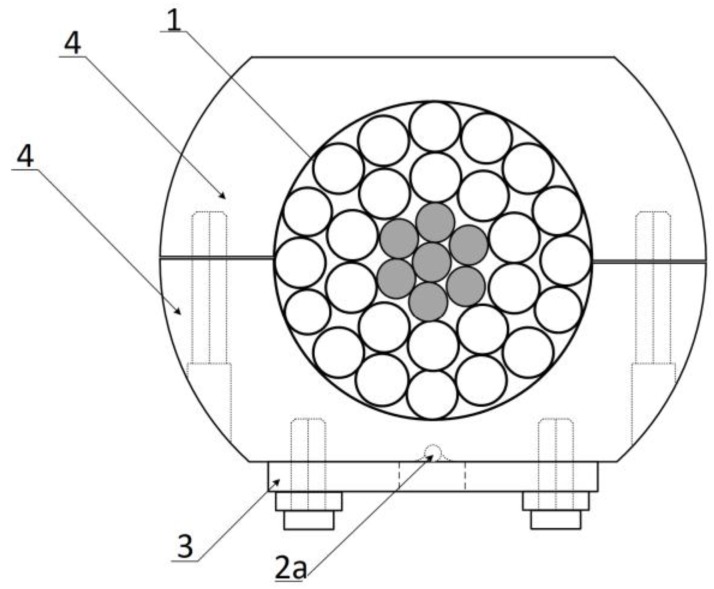
The cross-section of the sensing head of the power line sag measurement system where: 1, 2a, 3, and 4 are elements equivalent to those shown in Figure 2.

The idea of the whole optomechanical system is illustrated schematically in Figure 2 and the cross-section is shown in Figure 3. Figure 2b shows the proposed shape of the sensing plate which provides the non-uniform strain while the whole section is extended. The sensing grating was attached to the plate keeping the direction of the chirp shown in the figure below. Grating sections with the most extended periods are placed on the plate segment which is mostly elongated which provides the best response to the spectrum *FWHM* in the case of wire elongation.

The light from the light source (SLED) was directed to the CFBG sensor via an optical circulator. The optical spectrum reflected from the elongated CFBG was returned through the optical circulator to the optical spectrum analyzer (OSA). Thermal expansion and additional mechanical loads on the OTL wire cause variations in the length of the measured wire section surrounded by attached clamps as shown in Figure 2. In such cases, the response of the single-mode fiber with the inscribed CFBG results in the widening of the spectral characteristics which is caused by the nonlinearity of the strain applied to the transducer. 

The full-width half-maximum (*FWHM*) comparison of the spectral characteristics of the signals reflected from the Bragg sensor with the value assigned to the reference length of the segment gives information on the current elongation of the OTL wire. At the same time, changes in the wire surrounding temperature cause spectral shifts to the whole CFBG spectrum and do not affect the *FWHM*. The linear relationship between the elongation of the conductor, its temperature, and its stress allow for the determination of the elongation value. In the case of this sensor, the matrix equation of the processing can be written as:

(1) $$\left[\begin{array}{c} OP_{1}\\ OP_{2} \end{array}\right]=\left[\begin{array}{cc} SC_{11} & SC_{12}\\ SC_{21} & SC_{22} \end{array}\right]\times \left[\begin{array}{c} T\\ \Delta l \end{array}\right]$$

where *SC_ij_* (*i* = 1–2, *j* = 1–2) is the sensitivity coefficients that are defining the optical parameters sensitivity of the CFBG sensor *OP_i_* on the temperature *T* along with the elongation Δ*l*. Let us denote the matrix of coefficients *SC* as:

(2) $$M=\left[\begin{array}{cc} SC_{11} & SC_{12}\\ SC_{21} & SC_{22} \end{array}\right]=f(\Delta l,T)$$

The optical parameter *OP*_1_ denotes the full-width at half maximum (*FWHM*) of the measured CFBG spectrum, while the *OP*_2_ parameter reflects the spectral shift (*SSH*). Thus, it can be assumed that *OP*_1_ and *OP*_2_ depend upon the relative elongation Δ*l* and temperature *T* (Equations (3) and (4)):

(3) $$OP_{1}=f_{1}\left[\Delta l,T\right]$$

(4) $$OP_{2}=f_{2}\left[\Delta l,T\right]$$

In this case, we will look for the temperature values and maximum stress without knowing the value of matrix **M**. Let us denote the vector of the searching values as **sv**. This vector contains the value of the engineering strain *e* and the temperature *T* so that we can write:

(5) sv=[Δl,T]

The measurement values vector **mv** can be defined as follows:

(6) $$mv=[OP_{1},OP_{2}]=\left[FWHM_{CFBG},SSH_{CFBG}\right]$$

where *FWHM_CFBG_* is the full-width at half maximum of the *CFBG* spectrum and *SSH_CFBG_* denotes its spectral shift. Equation (1) can be written in the matrix form as follows:

(7) mv=Msv

The matrix **M** has 2 × 2 dimensions, is symmetrical for all vectors $x\in {\mathbb{R}}^{2}$, and is real. As a result of the measurements, the sensitivity matrix **M** is determined and the vector of searching values **sv** can be calculated based on the measured magnitude vector **mv**. Measuring the optical parameters of the *CFBG*, such as *FWHM_CFBG_* and *SSH_CFBG_* allows for the determination of the fiber temperature *T* and elongation ∆*l* according to the matrix equation below:

(8) $$\left[\begin{array}{c} OP_{1}\\ OP_{2} \end{array}\right]=\left[\begin{array}{cc} CSC_{11} & CSC_{12}\\ CSC_{21} & CSC_{22} \end{array}\right]\times \left[\begin{array}{c} T\\ \Delta l \end{array}\right]$$

where *CSC_ij_* are the complex sensitivity coefficients (*i* = 1, 2, *j* = 1, 2). The value ∆*l* which appears in Equation (8) is the relative elongation length measured between the clamps of sensing head (*l_ref_*) mounted on the conductor. 

The conductor sag *D* can be calculated as a function of Δ*l*, according to the reference conductor length *L_ref_*. The overhead transmission line conductor’s sag-tension calculations are typically based on the catenary equation, which describes an entirely flexible rope rigidly fixed at both ends. The catenary equation is defined using hyperbolic sine or cosine functions. However, it can be reliably approximated by a parabola. The main difference between a catenary equation and the parabolic approximation is that catenary assumes a constant weight per unit length through the conductor while the parabolic equation assumes an invariable weight per unit horizontal length. This simplification causes the sag calculation with the parabolic approximation to be smaller than when it is estimated with the catenary equation. 

The shape of a catenary is a function of the conductor weight per unit length weight *w*, the horizontal component of tension *H*, span length *S*, and the maximum sag of the conductor *D*. The exact catenary equation uses hyperbolic functions, as shown in Equation (9). The right side of the Equation (9) is an approximation of the hyperbolic cosine using the Maclaurin series expansion:

(9) $$y(x)=\frac{H}{w}\cosh\left(\left(\frac{w}{H}x\right)-1\right)=\frac{w\left(x^{2}\right)}{2H}.$$

For a flat span, the low point is at the center and the wire sag *D* is found by substituting *x = S/2*. Exact and approximate formulas for the sag calculations is shown in Equation (10):

(10) $$D=\frac{H}{w}\left(\cosh\left(\frac{wS}{2H}\right)-1\right)=\frac{wS^{2}}{8H}.$$

**Figure 4:**
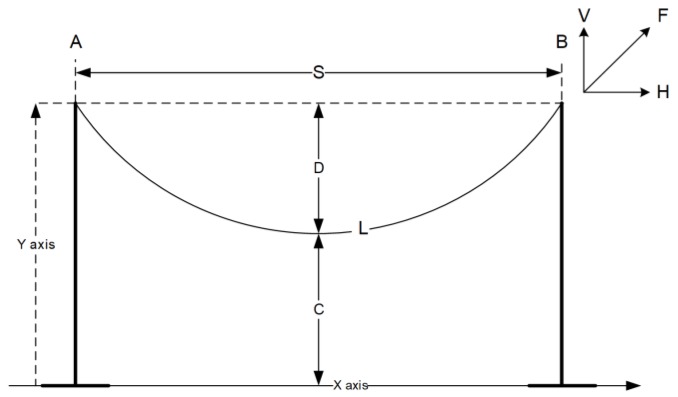
The conductor length, sag, clearance, and tension in a transmission line span.

The parabolic approximation is sufficiently accurate as long as the sag in the span does not exceed 5% of its length [36]. The power line sag *D* varies with the conductor temperature, ice, wind loading, and time as the conductor creeps. The horizontal tension *H* is equal to the conductor tension in the middle of the span shown in Figure 4. At the endpoints where the wire is fixed to the insulators, the conductor tension *F* is equal to the horizontal tension *H* plus the conductor weight per unit length *w* multiplied by the sag *D*, according to Equation (11) [36]:

(11) F=H+wD.

It is common to perform sag-tension calculations using only the horizontal tension component *H*, but the average of the horizontal and support point tension *F* is usually shown. The conductor length in the span can be calculated with the application of a catenary equation using Equation (12). The right side of Equation (12) corresponds to a parabolic approximation of the catenary function:

(12) $$L=\frac{2H}{w}\sinh\left(\frac{Sw}{2H}\right)=S\left(1+\frac{S^{2}w^{2}}{24H^{2}}\right).$$

The total conductor length can be expressed as a function of sag *D* as shown in the Equation (13):

(13) $$L=S+\frac{8D^{2}}{3S}.$$

Using Equation (13), we can write the formula for the conductor sag dependence *D* upon the span length *S* and the conductor length *L*, shown below:

(14) $$D=\sqrt{\frac{3S\left(L-S\right)}{8}}.$$

The difference between the conductor length *L* and span length *S* is defined as the conductor slack. Equation (14) shows that small changes in the slack give significant changes in the conductor sag. As it was mentioned above, conductor sag depends mainly on the total conductor length *L* when the span length *S* remains constant. The temperature dependency of the conductor length *L* is typically calculated using Equation (15):

(15) $$L_{2}=\alpha_{AS}\;L_{1}\;\left(T_{2}-T_{1}\right)+\beta \;L_{1}\;\left(\sigma_{2}-\sigma_{1}\right),$$

where index 1 and 2 are the beginning and end states, respectively; *L*_1_, *L*_2_: the conductor length in the span; *T*_1_, *T*_2_ are the conductor temperature; α_AS_ is the thermal elongation coefficient for ACSR; *σ*_1,_ *σ*_2_ are the conductor stress; and *β* and the wire elastic elongation coefficient. 

**Figure 5:**
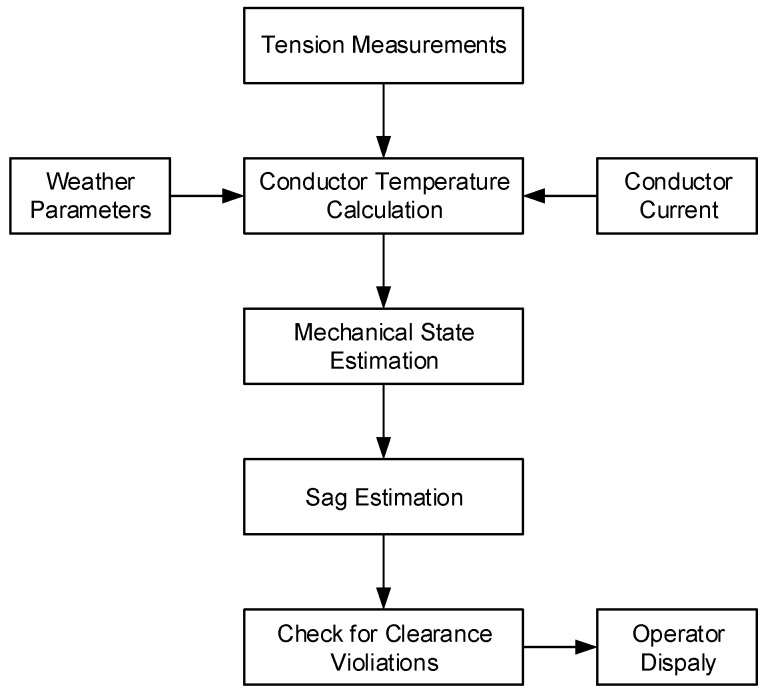
The sag estimation process reproduced from [26].

The typically temperature-tension calculation of the power line span is performed using Equation (16) [36] and solved using iterative methods:

(16) $$\sigma_{2}-\frac{S^{2}g_{2}^{2}}{24\beta \;\sigma_{2}^{2}}=\sigma_{1}-\frac{S^{2}g_{1}^{2}}{24\beta \;\sigma_{1}^{2}}-\frac{\alpha }{\beta }\left(T_{2}-T_{1}\right),$$

where *S* is the span length; *σ*_1_, *σ*_2_ = *H/A* is the wire stress; *g* = *w/A* is volumetric weight; *A* is the wire cross-sectional area; and *β* = *1/γ* is the wire elastic elongation coefficient. For ACSR 26/7 Hawk conductors, typically α = 18.7 × 10^−6^ 1/K, *γ* = 75,000 MPa, *A* = 276.2 mm^2^, *w* = 9.52 N/m, and g = 34.47 N/(m·mm^2^) [37]. In such cases, we need to measure tension, stress, or the wire temperature to estimate the total length of the wire in the span and then calculate sag. Sometimes, a calculation of the bare conductor temperature can be done using the methods presented in [28,29,30] based on weather parameters, electric current, or conductor tension measurements. The algorithm for conductor sag calculation was investigated by [26] and is presented in Figure 5. 

This paper introduces a direct method for calculation of the sag *D* based on a single spot conductor elongation measurement with a CFBG sensor. Assuming that the horizontal tension *H* and conductor thermal elongation is constant over the span, the relative elongation measurement Δ*l* of the selected 10 cm wire segment covered with the sensing head clamps provides the data required for the calculation of the total wire length *L*, as described with Equations (17)–(20). According to previous considerations, the relative wire elongation Δ*l* could be appointed as a function of the sensor *FWHM* spectral width as shown below:

(17) $$\Delta l=C_{\mathrm{OP}1}\cdot \Delta FWHM_{CFBG},$$

(18) $$E=\frac{\Delta l}{l_{ref}}=\frac{C_{\mathrm{OP}1}}{l_{ref}}\cdot \Delta FWHM_{CFBG},$$

where *E* is the relative elongation coefficient as a measured *FWHM* function; *C*_OP1_ is the experimentally evaluated elongation sensor sensitivity coefficient; *l_ref_* is the reference distance of the installed sensor clamps on the conductor; and Δ*l* is the elongation of the wire segment covered by the sensing head and measured by the CFBG sensor.

(19) $$L=L_{ref}\left(1+E\right).$$

Assuming the above (Equation (19)), the conductor sag dependence *D* (Equation (14)) we acquire the following form:

(20) $$D=\sqrt{\frac{3S\left(L_{ref}\left(1+E\right)-S\right)}{8}}.$$

According to Equations (14) and (19), it is shown that for OTL wire sag calculation is enough to measure an elongation of a particular section of the wire. 

## 3. Results

**Figure 6:**
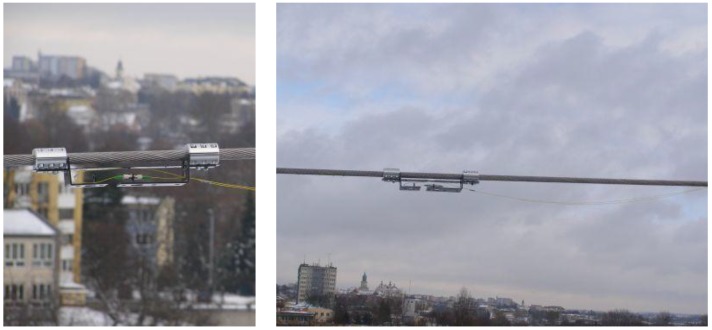
The CFBG based optomechanical system for the power line sag estimation in real conditions.

The surveys were performed on a real overhead power transmission line. The measurement system was mounted directly on a 110 kV OTL power line wire which was energized. The proper construction of the optomechanical system, provided its compact size and weight, makes it negligible to the mechanical workings of the power line. Figure 6 shows a photograph of the measurement system mounted on an ACSR 26/7 Hawk wire. 

**Figure 7:**
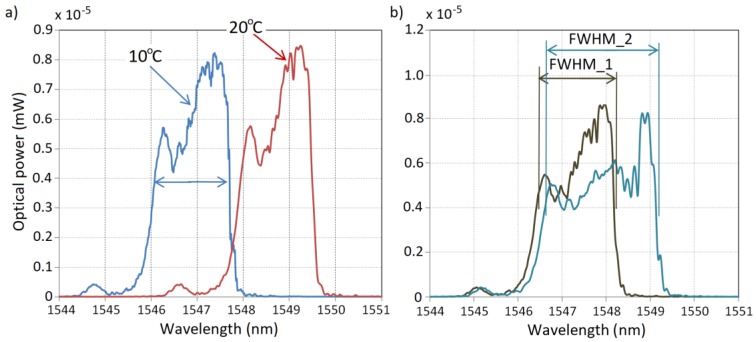
(**a**) a comparison of the spectra measured for the CFBG mounted in the proposed OTL wire monitoring system for 10 °C and 90 °C surrounding temperatures; (**b**) a comparison of the spectra measured for CFBG: FWHM_1 refers to the measurement directly after mounting the system on the wire and FWHM_2 refers to the measurement after 0.1% relative elongation.

The influence of temperature variations on the measurement system described above was measured for a short segment of the power line wire with the mounted complete system (clamps, steel plate with attached optical fiber). The OTL wire segment was placed in a climate-control chamber with a temperature range established at 10–90 °C. Figure 7a shows the spectral characteristics of the CFBG reflected signal measured for border cases of the surrounding temperature: 10 °C and 90 °C. The increase of the surrounding temperature of the wire changes the CFBG spectral characteristic by shifting the whole spectrum to longer wavelengths. The spectrum is not widened so the *FWHM* parameter of the sensor output characteristics has a quasi-constant character. However, the shift of the spectrum could be distinguished and measured and in this paper is called *SSH* (spectral shift). 

**Figure 8:**
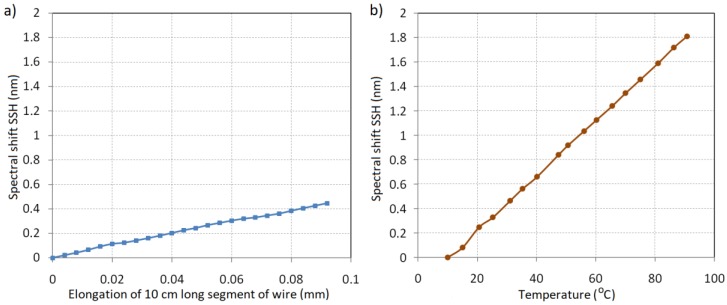
The comparison of *SSH* dependency on (**a**) the elongation of the testing plate; and (**b**) the changes of the CFBG sensor surrounding temperature.

In the case of chirped Bragg gratings, distinguishing the center wavelength is difficult because of the non-regular character of its spectra. In the processing characteristics shown in this paper, the *SSH* parameter is calculated based on the half spectral width of the appointed *FWHM*. Another situation occurs when the wire length changes due to the mechanical elongation which is shown in Figure 7b. The *FWHM* value sharply increases with the growth of the measured elongation, which promises that in the described measurement system it could be used as a determinant of the power line sag. Figure 8 shows the characteristics of the dependency of *SSH* on the wire elongation and the surrounding temperature variations in the selected ranges. 

**Figure 9:**
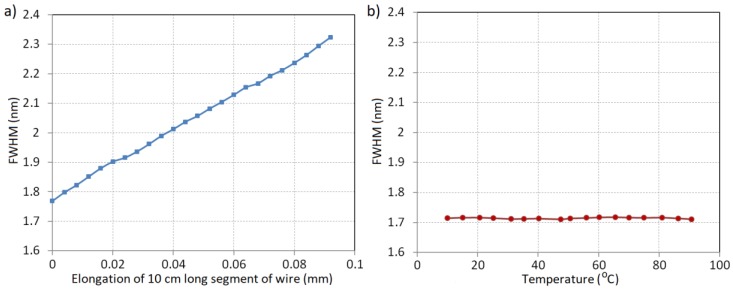
The characteristics of the *FWHM* spectral width dependency on (**a**) the relative elongation; and (**b**) the ambient temperature of the proposed system.

The graph depicted above shows that the spectral shift calculated as a half wavelength width of *FWHM* depends on both elongation and temperature. In the case of temperature variations, the transformation of the measured temperature to *SSH* has a linear character. The temperature sensitivity of the considered monitoring system configuration is associated with a thermal expansion coefficient of the material which the testing plate is made of along with the material of the OTL wire. The shift determined for the different elongation values results directly from the widening of the signal spectrum. As opposed to the *SSH* parameter, the *FWHM* spectral width depends only on the elongation of the measurement section and is practically insensitive to the temperature changes. Figure 9 shows the changing characteristics of the *FWHM* width dependent on the changes of elongation and temperature in the same ranges as in the case shown in Figure 8. 

According to Figure 9, the total wire elongation in the span and the wire sag can be anticipated as a function of *FWHM* and the initial state span parameters. Let us assume that the power line span is *S* = 300 m and constructed with ACSR 26/7 Hawk conductors that have a per unit length weight *w* = 9.75 N/m [37]. The mechanical characteristics of the span describe that, for a wire temperature of 10 °C, the horizontal tension is *H* = 14,600 N. Using Equation (12), it is then possible to calculate the reference wire length *L_ref_* (Equation (21)) and reference sag *D_ref_* (Equation (22)):

(21) $$L_{ref}=300\left(1+\frac{300^{2}\cdot 9.75^{2}}{24\cdot {14,600}^{2}}\right)=300.502\;m,$$

(22) $$D_{ref}=\sqrt{\frac{3\cdot 300\cdot (300.502-300)}{8}}=7.514\;m.$$

**Figure 10:**
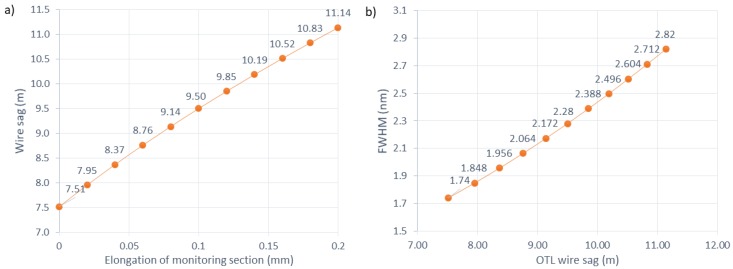
(**a**) The dependency of the OTL wire sag related with the elongation of the proposed measurement system plate calculated for the initial conditions described in Equations (21) and (22); and (**b**) the dependency of the measured *FWHM* for different sag values of the monitored OTL line.

**Figure 11:**
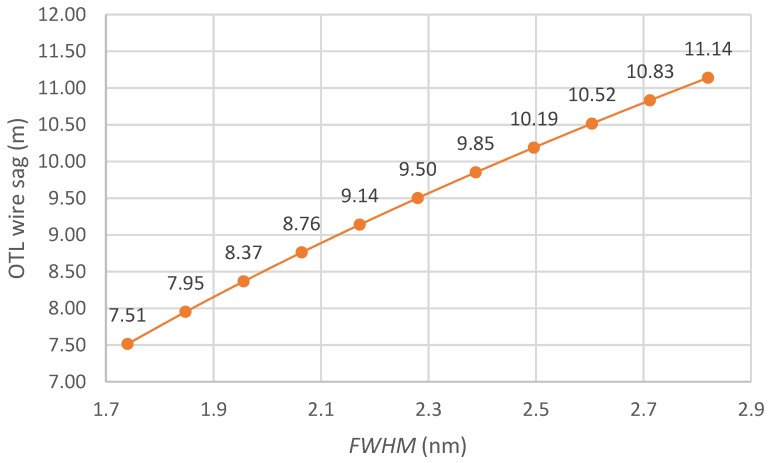
The dependency of the OTL wire sag as a function of the measured *FWHM.*

The initial conditions shown above are matched for an OTL span right after installing the wire on the poles. Weather conditions cause changes in the elongation of the wire due to icing, ambient temperature, wind speed and angle, and solar radiation. The elongation is also associated with the conductor temperature arising from the electric current flow. As a result of the total wire length *L*, changes in the power line sag *D* also vary. In the proposed optomechanical system, the variations of the length of the measured section affect the measured *CFBG_FWHM_* spectral width. Characteristics shown in Figure 10 and Figure 11 present how the power line sag will be influenced by the elongation of the testing OTL wire segment and how the *FWHM* spectral width is affected by sag changes of the power line with the initial parameters described by Equations (21) and (22). 

The coefficients *OP_1_* and *OP_2_* are two different parameters of the Bragg grating spectrum that will change due to the induced elongation or temperature changes. The matrix equation of the temperature and elongation sensor processing will take the form of Equation (8). Taking into account the results obtained from the measurements, it allows us to write the matrix equation in the form below:

(23) $$\left[\begin{array}{c} FWHM_{CFBG}\\ SSH_{CFBG} \end{array}\right]=\left[\begin{array}{cc} 0.00010 & 0.00603\\ 0.02261 & 0.00484 \end{array}\right]\times \left[\begin{array}{c} T\\ \Delta l \end{array}\right],$$

where *CSC_11_* is the sensitivity of *FWHM_CFBG_* to temperature (*CSC_11_* = 0.1 pm/K), *CSC_21_* is the sensitivity of *SSH_CFBG_* to the temperature (*CSC_21_* = 22.61 pm/K), *CSC_12_* is the sensitivity of *FWHM_CFBG_* to the elongation of testing section (*CSC_12_* = 6.03 pm/μm), and *CSC_22_* is the sensitivity of *SSH_CFBG_* to the elongation of the testing section (*CSC_22_* = 4.84 pm/μm). 

By analyzing Equation (23), it could be seen that the simultaneous measurement of the temperature and elongation is possible if, for the proposed monitoring system, we designate two different parameters of the CFBG which show different sensitivities for the measured quantities. It is only possible if the inequality condition *OP_1_* ≠ *OP_2_* is also met, which is fulfilled because *FWHM_CFBG_* ≠ *SSH_CFB__G_*. Analysis of Equation (23) allows us to conclude that with the known (determined experimentally) sensitivities of *FWHM_CFBG_* and *SSH_CFBG_* for the temperature and elongation, the measured quantities of *CSC_11_*, *CSC_21_*, *CSC_12_*, and *CSC_22_* could be determined simultaneously. The matrix of algebraic complements of all sensor sensitivities *CSC_11_*, *CSC_12_* along with *CSC_21_*, CSC_22_ have been calculated using Equation (24) and the received values have been shown in Equation (25):

(24) $$\begin{array}{l} d_{11}=(-1{)}^{1+1}\cdot CSC_{22}=CSC_{22},\;d_{12}=(-1{)}^{1+2}\cdot CSC_{21}=-CSC_{21}\\ d_{21}=(-1{)}^{2+1}\cdot CSC_{12}=-CSC_{12},\;d_{22}=(-1{)}^{2+2}\cdot CSC_{11}=CSC_{11} \end{array}.$$

By the condition of the non-zero matrix determinant of Equation (24), it is possible to construct its complementary matrix based on Equation (24), which we will now write in the form:

(25) $$\left[\begin{array}{c} T\\ \Delta l \end{array}\right]=\frac{1}{-0,00014}\left[\begin{array}{cc} 0.00484 & -0.00603\\ -0.02261 & 0.00010 \end{array}\right]\times \left[\begin{array}{c} FWHM_{CFBG}\\ SSH_{CFBG} \end{array}\right],$$

where *det*: the matrix determinant form of Equation (23) moreover, equals:

(26) $$det=CSC_{11}CSC_{22}-CSC_{21}CSC_{12}.$$

The fulfilled condition of the various sensitivity ratios of *FWHM_CFBG_* and *SSH_CFBG_* for the temperature and elongation gives the possibility of simultaneous determination of both quantities by measuring the spectrum shift (*SSH_CFBG_*) and the spectral width of the CFBG characteristic (*FWHM_CFBG_*). 

## 4. Discussion

The proposed method has a low cross-sensitivity, as seen in the final matrix of the processing system in Equation (25). An analytical determination of the OTL sag was proposed based on the CFBG spectra measurements mounted directly on the Hawk wire using the special semicircular screwed clamps forming the whole sensing head. Empirical studies have confirmed the low cross-sensitivity values (*CSC_11_* = 0.1 pm/K, *CSC_21_* = 22.61 pm/K, *CSC_12_* = 6.03 pm/μm and *CSC_22_* = 4.84 pm/μm). We have shown that such use of CFBGs and the suitable optomechanical system allows for the monitoring of the relative wire elongation and the OTL sag *D*. The presented method is non-sensitive to temperature changes (the *FWHM* width used for the determination of elongation is practically insensitive to thermal variations), provides repeatability of measurements (by the mechanical construction of clamps and the whole sensing head) and it is non-invasive . 

It is also worth mentioning that the design of the sensing head protects the optical fiber because changing the wire length within the possible elongation range does not damage the optical fiber. Additionally, the mass of the sensing head is negligible relative to the mass of the entire wire in the span so the sensor does not affect the object being measured. Figure 11 shows the dependency of the calculated sag of the power line with the assumed initial parameters on the measured *FWHM* spectral width. The potential of the method is increased by the use of a chirped fiber Bragg grating CFBG whose spectral characteristics are broader (more extensive) than in the case of a conventional FBG.

If the spectral response of the CFBG/FBG were to be measured using an interrogator (for example, based on an FBG optical filter) and not using an OSA, the measurement would be more accurate since the measured spectra would be broader and changes in its width would be measured more accurately by the interrogator.

## 5. Conclusions

In this article, we have presented a method that monitors the state of an OTL and measures its relative elongation and its actual sag. It is based on the new construction of CFBG sensors and is insensitive to the ambient temperature, the electromagnetic field and any other external typically influencing parameters. Insensitivity has been achieved by the structure of the sensing head that consists of clamps and the special monotonically, non-uniformly extendable steel plate that the CFBG is attached to. The proposed system can be non-invasively and directly embedded on the power line wire. The use of the Bragg grating with a linearly variable period allows for more considerable variation in the optical spectrum than standard FBGs, which translates into an increased sensitivity and accuracy. The obtained sensitivity of the *FWHM* width to elongation was 0.00603 nm/μm, and at the same time, the *FWHM* is practically insensitive to temperature changes (sensitivity coefficient reduced to 0.0001 nm/K). This condition allows for the determination of the wire elongation independently of temperature variations. In addition to the determination of the OTL wire extension, the proposed method also allows for the measuring of the temperature around of power line wire by meeting the conditions of the sensitivity matrix equation. 

Furthermore, the use of chirped fiber Bragg grating with a broad spectrum is advantageous because its spectrum is approximately ten times wider than the usual FBG. The measurements using a CFBG will be more precise than the standard FBG which has a much narrower range. Thus, the proposed system is entirely applicable in a configuration in which the spectra of the grating are interrogated with an optical filter (for example, a filter based on homogeneous FBG). Such a combination can be very interesting because it offers the possibility to eliminate the OSA and use a simple interrogator based on an optical filter.

The measurement point fixing system is designed for use for an indefinite time, which is of crucial economic importance. Additionally, such a designed system ensures the stability of its components on the OTL wire without any adverse effect on its strength, mainly due to the non-invasive fixing method. In the case of possible damages to the delicate components of the sensor system, it is possible to replace them with new ones with the same parameters. An important feature is the independence of the measurement from the influence of the outside temperature and the unavoidable electromagnetic field at the measurement site.

It should be noted that all determined sensitivity coefficients and final results are also dependent on the geometry of the transducer and the initial parameters of the CFBG sensor together with the control plate. Future work will involve an investigation into a sensitivity analysis for the function of changes of the parameters mentioned above and on the metrological conditions of the whole system.
